# Essential Oil from *Curcuma Longa* Leaves: Using Nanotechnology to Make a Promising Eco-Friendly Bio-Based Pesticide from Medicinal Plant Waste

**DOI:** 10.3390/molecules30051023

**Published:** 2025-02-23

**Authors:** Bianca Flexa-Ribeiro, Manoel D. N. Garcia, Ana Carolina de J. Silva, José Carlos T. Carvalho, Leandro Rocha, Silvia Maria M. Faustino, Caio P. Fernandes, Hellen F. da Silva, Francisco P. Machado, Lorane Izabel da S. Hage-Melim, Raimundo Nonato P. Souto, Gisele da S. Botas, Rodrigo A. S. Cruz

**Affiliations:** 1 Programa de Pós-Graduação em Ciências Farmacêuticas, Campus Marco Zero, Federal University of Amapá, Macapá 68903-419, AP, Brazil; bianca.flexa.ribeiro@gmail.com (B.F.-R.); karolcarolina06@gmail.com (A.C.d.J.S.); farmacos@unifap.br (J.C.T.C.); caio_pfernandes@yahoo.com.br (C.P.F.); lorane@unifap.br (L.I.d.S.H.-M.); rnpsouto@unifap.br (R.N.P.S.); giselebotas@gmail.com (G.d.S.B.); 2 Department of Biological and Health Sciences, Campus Marco Zero, Federal University of Amapá, Macapá 68903-419, AP, Brazil; m.d.juniorbio@gmail.com (M.D.N.G.J.); fitomathes@yahoo.com (S.M.M.F.); 3 Department of Pharmaceutical Technology, Faculty of Pharmacy, Fluminense Federal University, Niterói 24210-346, RJ, Brazil; leandromr@id.uff.br (L.R.); labnanofito@gmail.com (H.F.d.S.); fmachado@id.uff.br (F.P.M.)

**Keywords:** waste management, pest control, biopesticides

## Abstract

Nano-emulsions of essential oils (EO) and their chemical constituents are promising raw materials for the ecological control of *Tribolium castaneum*. *Curcuma longa* L. is a plant known for the properties of its rhizome, which is used in food, health, and hygiene products. Although its leaves are considered by-products with no commercial value, they produce an essential oil rich in bioactive monoterpenoids. This study aims to evaluate the repellency of nano-emulsions containing the EO from leaves of *C. longa* or its three main chemical constituents against *T. castaneum*. The representative mixture of EO extracted in four different months showed *p*-cymene (26.0%), 1,8-cineole (15.1%), and terpinolene (15.5%) as major compounds. Nano-emulsions of EO (HLB 16.7), terpinolene (HLB 15.0), 1,8-cineole (HLB15.0), and *p*-cymene (HLB 15.0) were repellent at concentrations of 11 μg/cm^2^ (EO, terpinolene, and *p*-cymene) and 1.1 μg/cm^2^ (1,8-cineole). The EO nano-emulsion droplet size increased linearly over time, remaining below 300 nm for 35 days. The EO nano-emulsion proved to be a green alternative to synthetic pesticides, as it was safe against the bioindicator *Chlorella vulgaris*. Furthermore, its main constituents were able to inhibit in silico the enzyme telomerase of *T. castaneum*, which is an enzyme essential for life. This study provides ideas for the utilization of EO from leaves of *C. longa* as raw material for new environmentally friendly plant-derived nanobiopesticides.

## 1. Introduction

One of the main challenges faced by industries is to design effective products that generate less stress on the environment and make better use of natural resources. A major part of industrial crop residues is constituted by the biomass that remains after using the valuable parts of plants, which can pollute the environment and harm human health. However, plant residue is not only an environmental problem but also an economic issue, as these materials still contain chemicals that could be useful raw materials for new products [1]. The industrial use of all organs of a plant is not common, which means that a large part of the biomass obtained at harvest is discarded without generating any resources. Although there are examples of the use of these residues for the manufacture of new products, a large amount of biomass containing bioactive chemical constituents remains as industrial waste. In summary, the development of new products from residues would have three main advantages: (i) obtaining raw material at a very low cost, (ii) no need to increase culture costs, and (iii) increasing the efficiency of the use of plant biomass, which is in line with the sustainable use of natural resources [2].

*Curcuma longa* L. is a plant species cultivated mainly due to the properties of its rhizome, which is a highly appreciated spice with pharmacological properties [3,4]. *Curcuma longa* should be harvested 120 days after planting and can be grown in all months of the year in tropical climates [5]. While extracts from the rhizome of *C. longa* are widely used in health and beauty products, including shampoos, body creams, and toothpastes, its leaves are considered a by-product with no industrial use. However, the leaves of *C. longa* are also a source of an essential oil rich in bioactive substances (mainly monoterpenes and monoterpene alcohols) that could be used as raw material for new value-added products [6,7,8]. Essential oils are mixtures of bioactive volatile natural products that can be used for pest control [9,10]. The anti-insect action of essential oil is associated with co-evolution between plants and phytophagous insects, which has exerted evolutionary pressure for millennia, leading to the development of a diversified chemical arsenal in plants. Essential oils and their constituents (usually terpenoids or phenylpropanoids) have shown potential for controlling *T. castaneum*, which is a secondary pest of stored grains that generates huge losses for the agroindustry [10]. In addition to grain consumption, which reduces commercial value, *T. castaneum* is a nomadic insect capable of carrying parasites that increase the risk of spreading resistance genes [11,12].

The high toxicity presented by synthetic pesticides used to control *T. castaneum* (mainly phosphine and methyl bromide) has led to the search for more sustainable and environmentally friendly ways to protect stored grains [13,14,15]. Natural substances with anti-insect action are green alternatives for pest control, as they are biodegradable compounds. In addition, they are naturally biosynthesized by the metabolism of living organisms without generating toxic chemical residues. Despite the insecticidal activity of traditional pesticides, the repellent activity of some natural products allows preventive control without using high concentrations that would be necessary to kill the insects. In addition, the use of such substances meets the increasing desire of consumers for ecologically sustainable products [16].

Although the potential of using essential oils as anti-insect agents is already well-reported, some difficulties (e.g., water solubility and prolonged stability) impair the development of a final product. The essential oil of *C. longa* leaves, for example, had its potential as an insecticide against *T. castaneum* demonstrated 20 years ago [17]. However, there were no advances to transform the oil into a raw material for a viable biotechnological product. In this context, the use of nanotechnology has been considered effective in overcoming the difficulties related to the development of bioproducts based on essential oils. Oil-in-water nano-emulsions are colloidal systems with a diameter below 300 nm, in which the oil is homogeneously dispersed in water [18]. Some advantages can be achieved by nano-emulsifying an essential oil, such as enhanced physical and chemical stability, protection against volatile loss, and improved bioactivity. These properties make nano-emulsification a promising strategy for the development of effective anti-insect agents.

The aim of the present study was to produce and evaluate the repellent action against *T. castaneum* of nano-emulsions containing the essential oil of *C. longa* leaves and its three main constituents.

## 2. Results and Discussion

### 2.1. Chemical Composition of the Essential Oil

Any biological activity is due to chemical and physical–chemical interactions between the active molecules and molecules present in the biological target. Thus, it is essential to know in depth the chemical composition of tested extracts. However, plants can vary their metabolism due to environmental factors and, therefore, increase or reduce the biosynthesis of certain metabolites. Therefore, in this work, collections and extractions were carried out in each quarter of the year and subsequently combined in equivalent proportions (25%), generating a sample of essential oil that represented the annual variations in its composition. The chemical composition of the essential oil is presented in Table 1.

The average yield of the hydrodistillations was 0.27 ± 0.05% (*w*/*w*). The essential oil obtained was mainly composed of monoterpenes with smaller percentages of oxygenated monoterpenes, with α-phallandrene (12.3), *p*-cymene (26.0%), 1,8-cineole (15.1%), and terpinolene (15.5%) being the most abundant. The results obtained corroborate data described in previous studies that suggest that there are differences between the essential oils of leaves and rhizomes of *C. longa*. Although it is well described in the literature that rhizome oil is rich in turmerones (sesquiterpene ketones), such metabolites are not commonly found in leaf oil [7,28]. This indicates that extracting essential oil from leaves is not just a way to get more of the same oil from the rhizome but rather a source of an oil with potentially different biological activity and mechanisms of action.

### 2.2. Preparation and Characterization of Nano-Emulsions

The nano-emulsification of oils in water can be achieved using several methodologies, including some with heating. The methodology to be chosen must consider the characteristics and limitations of the samples. As essential oils and their chemical constituents are volatile, in this work, a series of nano-emulsions was prepared using a solvent-free and low-energy method without heating to avoid losses by evaporation.

**Figure 1:**
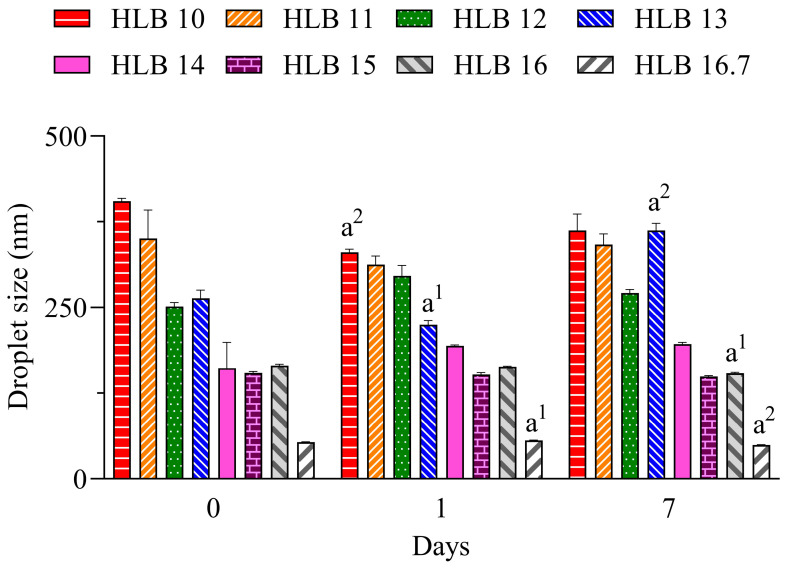
Diameter of droplets of *C. longa* nano-emulsions. a^1^ *p* < 0.05, a^2^ *p* < 0.01 in comparison to day 0. *n* = 3.

In Figure 1, it can be seen that the nano-emulsions prepared with *C. longa* essential oil and surfactants at HLB 14, 15, 16, and 16.7 presented droplet sizes below 200 nm during all evaluated periods. At the highest HLB value, which was reached solely with polysorbate 20, a lower size (~55 nm) was observed. The hydrodynamic size was analyzed by dynamic light scattering based on the Brownian movement of droplets, and overall, a size between 20 and 300 nm can be used to define a nano-emulsion [18].

**Figure 2:**
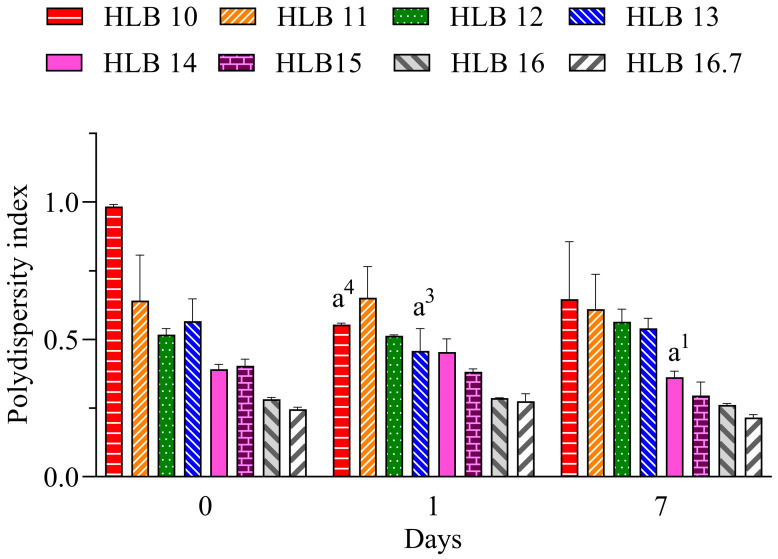
Polydispersity index of *C. longa* nano-emulsions. a^1^: *p* < 0.05, a^3^: *p* < 0.001, a^4^: *p* < 0.0001 in comparison to day 0. *n* = 3.

The polydispersity index (PdI) is related to the uniformity of droplet distribution. It can range from 0 to 1, where lower values (<0.6) have a tendency for more monodisperse distribution and increasing values (>0.7) have a tendency for polydisperse distribution, which is associated with large droplets or aggregated droplets [29]. Values below 0.25 suggest a narrow distribution that also can contribute to the long-term stability of nano-emulsions [30]. Therefore, considering the results for the nano-emulsions prepared with EO shown in Figure 2, the lowest PdI values are attributable to those prepared at HLB 16.7. Therefore, considering the better performance of the system prepared only with polysorbate 20, it can be suggested that the required HLB (rHLB) of the essential oil is 16.7.

**Figure 3:**
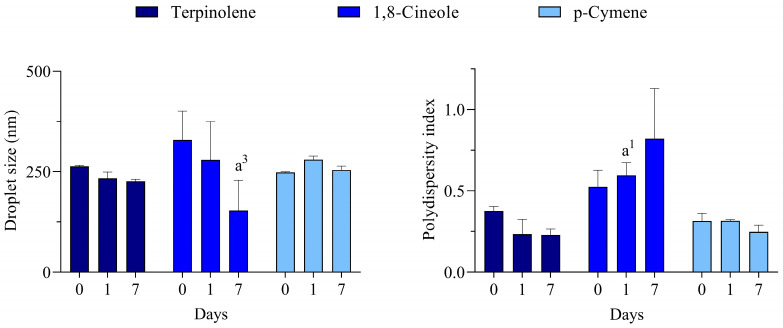
Droplet size and polydispersity index of nano-emulsions prepared with major constituents of *C. longa* essential oil. a^1^ and a^3^ represents *p* < 0.05 and *p* < 0.001 in comparison to day 0, respectively. *n* = 3.

For all monoterpenes, the most transparent/translucent systems for all days were reached with polysorbate 80. A more transparent aspect was observed for terpinolene and *p*-cymene, while the 1,8-cineole was slightly more translucent. Due to the macroscopical evaluation, in which most of the systems at a wide range of HLB values were opaque, the DLS analysis was carried out only for the polysorbate 80-based systems, and the results are presented in Figure 3.

Unlike some processes performed under high energy input, the low energy emulsification method used in this work only generates nano-emulsions when the conditions are most appropriate. According to the HLB concept proposed by Griffin [31], the best emulsions are formed when using a surfactant (or a mixture of them) in which the balance of the size and strength of the hydrophilic and lipophilic moieties of its molecule is the most suitable for emulsifying a given material. Therefore, it can be suggested that the rHLB of these monoterpenes is 15, which meets the HLB of the best surfactant used in the series.

Despite nano-emulsions being thermodynamically unstable systems due to unfavorable intermolecular interactions that occur in the oil/water interface, they can reach kinetic stability [32]. A known mechanism for the physical destabilization of nano-emulsions is Ostwald ripening [29]. This mechanism starts with the release of some partially soluble compounds by small droplets. These compounds pass through the external phase and are incorporated into large droplets in a continuous process that leads to complete destabilization. However, some complex oils have an alternative stabilizing mechanism, which is called Compositional Ripening. It was reported for essential oil-based nano-emulsions and relies on a tendency for maintenance of droplet composition [33]. In this context, the presence of several compounds in the complex *C. longa* essential oil may be contributing to the better system reached at HLB 16.5 since one would expect that a high HLB surfactant might also induce undesirable solubilization of compounds that might lead to Ostwald ripening. On the other hand, destabilization of nano-emulsions with pure terpenes would be triggered by this mechanism and, therefore, enough stable systems could only be reached with polysorbate 80 (HLB 15), which is slightly less hydrophilic than polysorbate 20 (HLB 16.7).

### 2.3. Stability of Essential Oil Nano-Emulsion

Although nano-emulsification increases the kinetic stability of oil-in-water mixtures, the system remains thermodynamically unstable with a tendency to break down over time. Therefore, it is important to verify the stability of the produced nano-emulsions.

A new nano-emulsion was prepared with the essential oil to evaluate its stability. As can be seen in Table 2, the droplet size increased significantly up to day 21. After remaining stable, the droplets decreased again steadily from day 50 until the end of the experiment on day 80. The polydispersity index data corroborate the hypothesis that there was a growing disorganization of the nano-emulsion system. As with droplet size, the polydispersity index varied gradually during the experiment.

**Figure 4:**
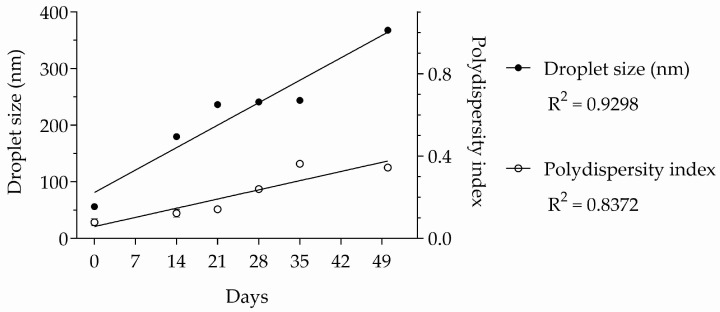
Linear regressions of droplet size and polydispersity index of essential oil nano-emulsion over time. *n* = 3.

After the analysis of variance (ANOVA) described in Table 2, the data were subjected to a subsequent analysis using linear regression to test the linearity between the physicochemical properties and time. Both droplet size and the polydispersity index varied linearly with time (R^2^ = 0.9298 and 0.8372, respectively), as shown in Figure 4.

Due to their thermodynamic instability, it is already expected that the physicochemical properties of nano-emulsions change as a function of time. Thus, the greatest importance of a stability study of these products is to verify the time in which they maintain their physicochemical properties within the limits used to define a nano-emulsion.

The first days after preparation of a nano-emulsion are critical for its stability, since freshly prepared formulations may experience a marked deterioration in their physicochemical properties. Therefore, many studies on the preparation of nano-emulsions monitor their stability for up to 30 days [34,35,36]. The formulation prepared in the present study remained as a nano-emulsion until the 35th day after preparation, which demonstrates good stability. However, future commercial use of this nano-emulsion will depend on more in-depth studies aimed at increasing its shelf life.

There is no consensus on the amount of time considered adequate for studies on the stability of nano-emulsions. Thus, there is great variation in the analysis times for the physicochemical properties of the nano-emulsions. While some studies track formulations for months, others publish only analysis results for freshly prepared emulsions. [19,37]. The data obtained in this work show good stability for this first nano-emulsion of the essential oil of *C. longa* leaves.

### 2.4. Repellency Assay

Essential oils, as well as many other plant metabolites, are products of millenary co-evolution between plant species and their natural enemies [38]. This is one of the reasons why essential oils and their constituents are known antimicrobial and anti-insect agents, acting as larvicides, insecticides, or repellents against the latter [9]. Volatile substances also have a great advantage over non-volatile metabolites, such as alkaloids and polyphenols: they can spread in the air and prevent damage to the plant, acting as protective agents. This property is very useful for the integrated management of pests, in which it is intended to avoid the degradation of plants of commercial interest, causing the least possible damage to the environment and human populations. In this context, essential oils are good candidates for raw material for the development of biochemical biopesticides, which are defined by the United States Environmental Protection Agency (EPA) as naturally occurring substances that control pests by non-toxic mechanisms.

To classify the repellent activity, not only the repellency percentage value was used but also the statistical criterion previously described by Lima and coworkers, in which samples were only considered repellent when positive repellency percentage values were significantly different from the maximum value of those considered inactive (0.1%) [19]. The results are described in Table 3.

Although the repellent activity classification of some samples changed between 2 and 4 h, there were no significant changes between the repellency values observed in the first and second readings (*p* > 0.05). This indicates that the nano-emulsions quickly reached maximum repellency and managed to keep it unchanged until the end of the experiment.

The essential oil showed repellent activity up to a concentration of 11 μg/cm^2^, which is in accordance with those of samples considered significantly active in previous works [40,41]. These results can be explained by the chemical composition of the essential oil, which is rich in substances known to be active against *T. castaneum*. The action of monoterpenes against insects is widely known [42]. The three most abundant compounds in the essential oil, terpinolene, 1,8-cineole, and *p*-cymene, have previously demonstrated repellent activity against *T. castaneum*. In fact, as can be seen in Table 1, approximately 71% of the representative mixture of the oil is composed of repellent compounds. However, this is the first time that nano-emulsions of terpinolene, 1,8-cineole, and *p*-cymene have been tested against this insect.

The *p*-cymene and terpinolene nano-emulsions showed repellent activity at the same concentrations as the essential oil. The 1,8-cineole nano-emulsion maintained its repellent activity even at the lowest tested concentration (1.1 μg/cm^2^). The literature shows that 1,8-cineol is a monoterpenoid with high repellent activity, which was also verified for its nano-emulsion in this work [23].

### 2.5. Ecotoxicity Assay

Pollution associated with pesticide accumulation is an emergent global problem [43]. Even low-toxicity products applied in low concentrations can be leached by rain and accumulate in aquatic environments, causing pollution and environmental imbalance. Ecotoxicity tests are useful tools to assess the effects of synthetic or natural pesticides that enter the environment. Algae, such as *Chlorella vulgaris*, are important bioindicators, as they form the basis of many aquatic food chains and are sensitive to the presence of pollutants in water [44].

The ecotoxicity test was based on comparing the cell density of *Ch. vulgaris* in the presence or absence of the essential oil nano-emulsion. The lowest active concentration of the nano-emulsion against *T. castaneum* was chosen as the basis for the ecotoxicity test. However, as the repellency test takes place on a two-dimensional surface, a calculation was necessary to define a proportional concentration in the three-dimensional aquatic model of ecotoxicity. Considering that at a concentration of 11 μg/cm^2^, there are 3.31 μg for each spatial dimension, the proportional three-dimensional concentration used was 36.5 μg/cm^3^. The results obtained are described in Table 4.

There were no significant differences between the cell densities in the groups on any of the days (*p* > 0.05). In addition, the comparison of the area under the curve values showed that there were no significant differences between the total amounts of cells found in both groups throughout the experiment. The results indicate that the nano-emulsion has a low potential to cause environmental impacts on water ecosystems.

### 2.6. Molecular Docking

The bioactivity of compounds is ultimately the result of physicochemical interactions between their molecules and key molecules in target organisms. In silico molecular docking analysis allows the evaluation of ligand–receptor interactions, helping to explain the activity presented in in vivo or in vitro assays. Telomerase is a ribonucleoprotein responsible for replicating the ends of chromosomes and maintaining the genome in its integrity, basically consisting of a reverse transcriptase, or catalytic unit, and a non-coding RNA molecule [45,46,47]. Its inhibition implies the shortening of the telomere sequence, which triggers several effects, such as senescence or apoptosis of several cells [45].

The RMSD value obtained in the target validation step (PDB ID: 5CQG) was 0.678 Å. RMSD values below 2 Å are more accepted since they indicate that a correct fit of the ligand occurred in a favorable spatial orientation in the protein [48]. The origin coordinates obtained in validation for this target were x = 22.08, y = 7.22, z = −31.75 at a radius of 10 Å.

According to the coordinates obtained in the validation, the binding site was defined to start the docking with the molecules under study (terpinolene, 1,8-cineole, and *p*-cymene). From this, it was possible to evaluate the interactions, types of interactions, the distances of each one, and the amino acid residues involved in the interactions and which atom or region each one is interacting with. The results are shown in Table 5.

**Figure 5:**
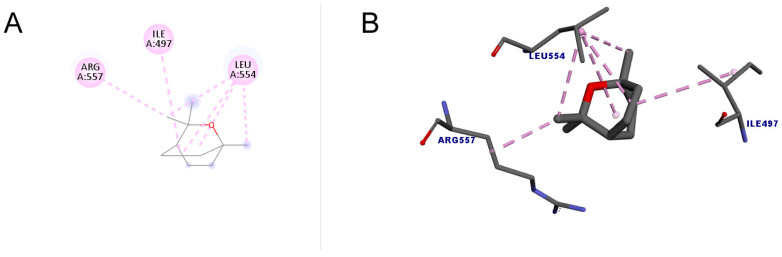
Molecular docking of 1,8-cineole in the telomerase target. (**A**) Two-dimensional model; (**B**) three-dimensional model. Intermolecular interactions of the alkyl type are represented by the light pink color.

Figure 5 shows the docking between 1,8-cineole and the enzyme telomerase. A total of six interactions occurred, all of which were hydrophobic of the alkyl type, that is, interactions that occur between alkyl groups. It is noteworthy that of the six bonds, four were interactions with the amino acid LEU554. The docking score value was 34.01.

**Figure 6:**
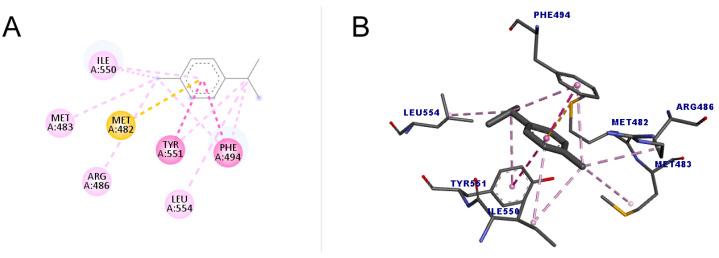
Molecular docking of *p*-cymene in the telomerase target. (**A**) Two-dimensional model; (**B**) three-dimensional model. Intermolecular interactions are represented by colors: pi–sulfur bonds (yellow color), pi–pi stacked and pi–pi T-shaped, (darker pink color), and alkyl and pi–alkyl (light pink color).

As shown in Figure 6, *p*-cymene performed better compared to 1,8-cineole both in the number of interactions and the score value (48.76). All interactions were hydrophobic, with one pi–sulfur, one pi–pi stacked, one pi–pi t-shaped, four alkyl, and four pi–alkyl, totaling eleven interactions. The pi–sulfur interaction occurred between the residue Met482 with the aromatic group of *p*-cymene, of the order sp^2^. The pi–pi stacked connection is a type of interaction that occurs between aromatic groups, containing a pi orbital, which was detected between the Phe494 residue and the ligand. As for the pi–pi T-shaped type, which occurred with Tyr551, it is an interaction that also occurs in cyclic systems with double bonds, which form a specific angle for each ring in a spatial configuration that has the T format [49].

**Figure 7:**
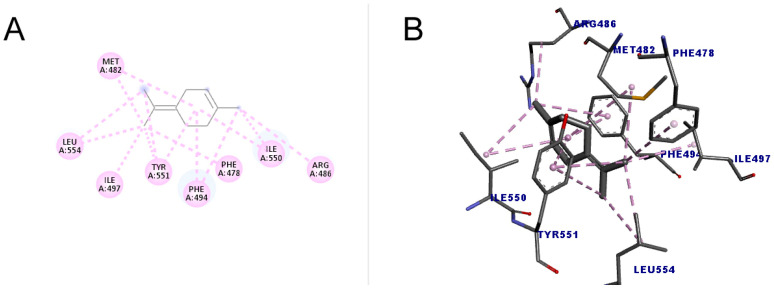
Molecular docking of terpinolene in the telomerase target. (**A**) Two-dimensional model; (**B**) three-dimensional model. Intermolecular interactions of the alkyl and pi–alkyl types are represented by the light pink color.

The molecule with the highest number of interactions and the highest score (49.15) was terpinolene (1-methyl-4-propan-2-ylidenecyclohexene), which performed 14 interactions with the amino acid residues of two types, alkyl and pi-alkyl interaction, indicating that due to these characteristics, it may have a greater inhibitory action when compared to other substances. The results are shown in Figure 7.

In a previous study that performed docking between telomerase obtained from *T. castaneum* and a low molecular weight inhibitor (BIBR1532), it was possible to identify the region where the ligand is located and that inhibits telomerase activity [50]. The result of this study demonstrated that the ligand was located in the RNA binding domain (TRBD). The presence of residues Phe478, Val491, Tyr551, and Leu554 in this region allowed greater stability in the orientation of the ligand around the site, from the formation of the so-called hydrophobic “pocket”. In addition to these amino acids, other residues line the inside of this pocket, such as Met482, Met483, lle497, Trp449, and Ile550. Even though the BIBR1532 molecule did not induce a marked change in conformation, these interactions allowed a more favorable and firm position of the ligand in the site, causing an inhibitory action on the enzyme.

According to the results obtained in this work, it can be inferred that the tested monoterpenes are capable of interacting with amino acid residues similar to those of a known telomerase inhibitor (BIBR1532), which suggests that these structures may have potential inhibitory activity on the enzyme from *T. castaneum*.

## 3. Materials and Methods

### 3.1. Plant Material

*Curcuma longa* rhizomes were obtained from a local producer and cultivated outdoors in the city of Macapá, Brazil (0°01′24″ N 51°09′31″ W), exclusively for this study. A voucher specimen was used for the botanical identification and deposited in the herbarium of the Instituto de Pesquisas Científicas e Tecnológicas do Estado do Amapá (IEPA), Macapá, AP, Brazil, under the registration code 019430. In February, May, August, and November 2020, a fraction of the cultivated plants was harvested. In February and May, the plants were in flower, while in August and November they were not. The leaves were separated from the rest of the plant using pruning shears and immediately subjected to hydrodistillation.

### 3.2. Chemicals

Analytical grade acetone was purchased from Sigma-Aldrich (São Paulo, SP, Brazil), terpinolene (≥95%, purity) and *p*-cymene (≥97% purity) were purchased from Merck (São Paulo, SP, Brazil), and 1,8-cineole (97% purity) was purchased from Quinarí (Ponta Grossa, PR, Brazil). The surfactants sorbitan monooleate, polysorbate 80, and polysorbate 20 were obtained from Sigma-Aldrich (São Paulo, SP, Brazil). All other chemicals were obtained from Synth (Diadema, SP, Brazil).

### 3.3. Extraction and Determination of the Chemical Composition of the Essential Oil

The fresh leaves (700 g) were ground in a blender with distilled water (3 L) and transferred to a 5 L flask connected to a Clevenger-type apparatus containing a water-cooled receiver to avoid evaporation losses. After the extractions, the essential oils from different months were centrifuged, dried with anhydrous sodium sulfate, and stored at −17 ± 1 °C. They were individually analyzed and used for the preparation of a representative mixture (25% of each oil) for nano-emulsion preparation and biological assay.

Qualitative gas chromatography (GC) analysis was performed using a GC-MS-QP2010 gas chromatograph equipped with a ZB-5 capillary column (i.d. = 0.25 mm, length 30 m, film thickness = 0.25 µm) and coupled to a mass spectrometer (MS) (Shimadzu, Barueri, SP, Brazil) using electron ionization at 70 eV (1 scan/s). The column temperature was programmed as follows: 60 °C for 5 min and increased at 3 °C/min until 290 °C. The sample was diluted in chloroform (1:100, *v*/*v*), injected into the column, and carried by helium at a flow rate of 1 mL/min (split injection ratio 1:40). The retention indices were calculated by the interpolation of each substance’s retention time and the retention time of a mixture of aliphatic hydrocarbons analyzed in the same conditions [51]. The identification of the essential oil constituents was performed by comparison of their arithmetic indices and mass spectra with literature data [27]. Quantitative analysis of the chemical constituents was performed using the peak area normalization method using a GC coupled to a flame ionization detector (FID) (Shimadzu, Barueri, SP, Brazil). Conditions were the same as the GC-MS analysis with the exception of the carrier gas, which was hydrogen in the GC-FID.

### 3.4. Determination of the Required Hydrophile–Lipophile Balance (rHLB) of C. Longa Essential Oil and Its Major Constituents

A series of emulsions containing the representative mixture of the essential oils of *C. longa* and its main constituents terpinolene, 1,8-cineole, and *p*-cymene were prepared at various HLB values (10 to 16.7) using the non-ionic surfactants sorbitan monooleate (HLB 4.3), polysorbate 80 (HLB 15.0), or polysorbate 20 (HLB 16.7). The resulting HLB values were obtained by performing binary mixtures of surfactants in proportions determined using the equation rHLB = [(HLB_A_ × m_A_) + (HLB_B_ × m_B_)]/(m_A_ + m_B_), in which HLB_A_ and HLB_B_ represent the HLB values of each surfactant. The mass (g) of each surfactant is represented by m_A_ and m_B_.

The required hydrophile–lipophile balance (rHLB) value of each oil was determined as the HLB value capable of producing the nano-emulsion with the best macroscopic and physicochemical properties, remaining stable for 7 days.

### 3.5. Preparation of the Nano-Emulsions

The nano-emulsions were produced using a low-energy method. First, the essential oil or its major constituents were mixed with surfactant at a specific rHLB value, affording the oily phase (2.5%, *w*/*w*). Distilled water was added dropwise under continuous stirring until a final mass of 2.0 g.

### 3.6. Characterization of the Nano-Emulsions

The nano-emulsions had their droplet size distribution evaluated by dynamic light scattering (DLS) at 90° using the Nano ZS (Malvern Instruments, Malvern, UK) equipped with a 10 mW “red” laser (X = 632.8 nm). Each nano-emulsion was analyzed immediately after preparation (day 0) and after 1 and 7 days of storage at room temperature. All measurements were performed in triplicate. Data were expressed as the mean ± standard deviation. Additionally, the essential oil nano-emulsion was submitted to a stability analysis, being analyzed on days 0, 14, 21, 28, 35, and 50.

### 3.7. Insects

Adults of *T. castaneum* of about 7 days of age were used in the experiments. The insects were reared and maintained in the Arthropod Laboratory of the Federal University of Amapá with controlled temperature (28–30 °C) and relative humidity (70–80%). The insects were reared in a growth chamber at 28 °C, 60% relative humidity and fed wheat flour with 12–13% humidity mixed with yeast (10:1, *w*/*w*).

### 3.8. Repellency Assay Methodology

The repellency contact assay was carried out according to [19]. Whatman filter papers with a diameter of 8.5 cm were sub-divided through a line into two equal sections identified as “test” and “control”.

Then, the filter papers were placed on Petri dishes and impregnated with samples at the following concentrations: 176; 88; 44; 11; and 1.1 µg/cm^2^ (*n* = 5). For the control, formulations were prepared under the same conditions as the nano-emulsions but without the addition of essential oil or terpenes. After 10 min, 20 *T. castaneum* were placed around the centers of the plates, which were immediately covered with a fabric that allowed the exchange of vapors. After 2 h and 4 h, the individuals in the test section were counted and the percentage of repellency was calculated as follows:

(1) PR=[(C−T)/(C+T)]×100

where PR = percentage of repellency; C = number of insects in the control section; T = number of insects in the test section.

The repellent action of the five concentrations was classified according to the respective PR: class 0 (−0.1% to 0.1%), class I (0.1 to 20%), class II (20.1 to 40%), class III (40.1 to 60%), class I (60.1 to 80%), or class V (80.1 to 100%) [39]. Sample concentrations with a mean PR value below −0.1 were considered attractive if statistically different from 0.1%, while those with a mean PR above 0.1% were considered repellent if statistically different from −0.1%. The absence of statistically significant differences within these value limits was considered inactive.

### 3.9. Acute Toxicity Against Chlorella Vulgaris

*Chlorella vulgaris* was reared at the Laboratory of Algae Cultivation (Federal University of Amapá). The overall conditions of cultivation of the green algae included an aqueous solution of nitrogen/phosphorus/potassium (NPK, 15:05:05), a constant photoperiod (2000 Lux), and a temperature of 24 ± 1 °C.

The samples were solubilized in 50 mL of the medium at a concentration of 36.5 μg/cm^3^. The culture media of the control and test groups were prepared with the same cell density (approximately 30,000 cells/mL). After 1, 2, and 3 days, the cell densities were measured for the control (non-treated) and samples in quintuplicate.

The cells were counted in a Neubauer chamber coupled to an optical microscope Quimis^®^ (Diadema, SP, Brazil) with the addition of lugol for better visualization. Cell growth inhibition was determined by comparing cell density values between the test and control groups.

### 3.10. Molecular Docking Methodology

Terpinolene, 1,8-cineole, and *p*-cymene ligands were optimized with the HyperChem 8.0 software using the semi-empirical method of the RM1 type (Recife model 1) [52]. The GOLD 2020.1 program (Genetic Optimization for Ligand Docking) was used to predict the best interactions between the ligand and the target [53].

The crystal structure of the telomerase protein of the organism *T. castaneum* was obtained from Protein Data Bank (PDB) database (PDB ID: 5CQG, 2.30 Å resolution, complexed with BIBR1532 inhibitor) [50].

### 3.11. Statistical Analysis

Analysis of variance (ANOVA) followed by Tukey’s test and linear regression were conducted using the Software GraphPad Prism 8.4.3 (San Diego, CA, USA). Differences were considered significant when *p* < 0.05.

## 4. Conclusions

The preparation of nano-emulsions containing the essential oil from leaves of *Curcuma longa* using methods with low energy input and without heating proved again to be a valid strategy to prepare stable and effective oil-in-water systems. The repellent nano-emulsions containing the essential oil of *C. longa* leaves proved to be a green alternative to synthetic pesticides, as it was safe against the bioindicator *Chlorella vulgaris*. Furthermore, the leaves of *C. longa* are by-products of obtaining the rhizome, which makes it an abundant material, with low cost and low environmental impact. The analysis of the chemical composition of the essential oil allowed the study of the isolated action of each of its major constituents. Terpinene, 1,8-cineole, and *p*-cymene nano-emulsions were repellent at the same concentrations as the oil, with 1,8-cineole being active at an even lower concentration. The action of the major monoterpenes against *T. castaneum* may be, at least in part, related to their ability to interact with that insect’s telomerase enzyme, especially terpinolene, which presented better results when compared to the other two compounds. However, the potential of a raw material depends on the balance between its advantages and disadvantages. Therefore, studies focused on improving the nano-emulsion and evaluating its economic viability are necessary.

## Figures and Tables

**Table 1:** Chemical composition of the essential oil from leaves of *Curcuma longa*.

Compound	Percentage Area (%)					RI_C_	RI_L_	Repellency Against *T. castaneum*
	February	May	August	November	RM			
α-Pinene	4.0	2.2	2.7	3.8	3.2	934	932	RA [19]
Sabinene	0.5	0.5	0.4	0.7	0.5	973	969	RA [20]
β-Pinene	5.7	3.5	2.6	5.5	4.4	980	974	RA [19]
Myrcene	1.2	1.6	1.6	1.4	1.4	988	988	NI
δ-2-Carene	0.0	0.4	0.3	0.3	0.2	998	1001	NI
α-Phellandrene	1.9	15.0	24.4	4.4	12.3	1008	1002	NI
δ-3-Carene	1.5	1.3	1.2	1.7	1.4	1011	1008	RA [21]
α-Terpinene	0.0	0.8	1.1	0.0	0.5	1018	1014	RA [22]
*p*-Cymene	34.8	19.7	13.0	37.2	26.0	1025	1020	RA [22]
Limonene	2,3	2.4	1.9	3.2	2.5	1030	1024	RA [19]
1,8-Cineole	19.6	11.4	10.7	19.3	15.1	1033	1026	RA [23]
(*E*)-β-Ocimene	0.0	0.2	0.0	0.0	0.9	1045	1044	NI
γ-Terpinene	0.0	0.9	1.1	0.0	0.5	1058	1054	RA [20]
Terpinolene	0.8	25.2	29.5	1.4	15.5	1087	1086	RA [24]
*p*-Cymenene	1.0	0.3	0.3	0.6	0.5	1092	1089	NI
Linalool	1.7	0.5	0.5	1.0	0.9	1100	1095	RA [21]
cis-Thujone	0.0	0.2	0.0	0.0	0.1	1104	1101	NI
Trans-Dihydro-β-Terpineol	0.5	0.0	0.0	0.0	0.1	1132	1134	NI
(*E*)-Epoxy-Ocimene	4.0	1.1	0.5	3.5	2.2	1144	1137	NI
Borneol	0.5	0.3	0.0	0.0	0.2	1175	1155	RA [25]
Terpinen-4-ol	0.8	0.3	0.3	0.4	0.4	1182	1174	RA [26]
*p*-Methyl-Acetophenone	1.3	0.0	0.0	0.0	0.3	1187	1179	NI
*p*-Cymen-8-ol	9.7	2.1	1.2	6.3	4.5	1188	1179	NI
*o*-Cumenol	1.2	0.5	0.5	0.9	0.8	1197	1196	NI
γ-Terpineol	1.2	1.9	1.8	0.9	1.4	1205	1199	NI
Monoterpenes	53.8	74.0	80.1	60.3	69.9			
Oxygenated Monoterpenoids	35.1	17.0	15.0	28.6	23.5			
Total	94.2	92.3	95.6	92.5	96.0			

RI_C_—calculated retention index, RI_L_—retention index, reported from previous work [27], RA—compound with previously reported repellent activity, NI—compound with no information on repellent activity.

**Table 2:** Stability of nano-emulsion of essential oil from *Curcuma longa* leaves.

Days	Droplet Size (nm)	Polydispersity Index
0	56.0 (±1.758) a	0.078 (±0.018) a
14	179.8 (±0.651) b	0.122 (±0.020) b
21	236.4 (±3.113) c	0.142 (±0.007) b
28	241.0 (±0.751) cd	0.240 (±0.004) d
35	243.8 (±2.996) d	0.362 (±0.009) e
50	367.9 (±1.721) e	0.345 (±0.007) e

Data are expressed as mean values ± SD of three replicates. *n* = 5. Mean values within a column sharing the same letters are not significantly different (*p* < 0.05).

**Table 3:** Repellency of the essential oil from leaves of *C. longa* and nano-emulsions against *T. castaneum*.

Sample	Concentration	Percentage Repellency (%) ^x^		Classification ^y^	
	(µg/cm^2^)	2 h	4 h	2 h	4 h
Essential oil	176	74.0 (±15.1)	88.0 (±13.0)	Class IV	Class V
nano-emulsion	88	76.0 (±11.4)	84.0 (±13.4)	Class IV	Class V
	44	70.0 (±10.0)	82.0 (±8.3)	Class IV	Class V
	11	70.0 (±29.1)	80.0 (±12.2)	Class IV	Class IV
	1.1	0.0 (±25.4)	6.0 (±20.7)	Inactive	Inactive
Terpinolene	176	76.0 (±19.4)	74.0 (±13.0)	Class IV	Class IV
nano-emulsion	88	64.0 (±30.0)	76.0 (±15.1)	Class IV	Class IV
	44	42.0 (±33.4)	46.0 (±16.7)	Class III	Class III
	11	48.0 (±37.0)	40.0 (±33.9)	Class III	Class II
	1.1	5.0 (±13.0)	0.0 (±12.2)	Inactive	Inactive
1,8-cineole	176	72.0 (±22.8)	83.2 (±10.0)	Class IV	Class IV
nano-emulsion	88	72.0 (±34.9)	78.0 (±8.3)	Class IV	Class IV
	44	72.0 (±21.6)	64.0 (±27.7)	Class IV	Class IV
	11	74.0 (±11.4)	71.0 (±4.4)	Class IV	Class IV
	1.1	44.0 (±11.4)	48.0 (±16.4)	Class III	Class III
*p*-cymene	176	74.0 (±29.6)	66.0 (±35.0)	Class IV	Class IV
nano-emulsion	88	56.0 (±18.1)	62.0 (±19.2)	Class III	Class IV
	44	48.0 (±24.47)	45.0 (±20.9)	Class III	Class III
	11	42.0 (±44.4)	41.0 (±37.6)	Class III	Class III
	1.1	0.0 (±13.0)	4.0 (±11.4)	Inactive	Inactive

^x^ Values expressed as means ± SD (*n* = 5). ^y^ Classification of repellent activity according to McDonald and coworkers [39].

**Table 4:** Viable cells of the green microalgae *Chlorella vulgaris* subjected to the nano-emulsion of the essential oil from leaves of *Curcuma longa* (expressed in oil content).

Day	Cell Density (Cell/mL)		Statistical Analysis
	Control	Nano-Emulsion	
0	30,000 (±3535.5)	31,000 (±5477.2)	Not significant
1	27,000 (±9746.8)	35,000 (±19,685.0)	Not significant
2	35,000 (±13,693.0)	29,000 (±15,165.8)	Not significant
3	26,000 (±13,416.4)	18,750 (±7500.0)	Not significant
AUC	90,000 (±13,761)	89,000 (±18,080)	Not significant

AUC—Area under the curve. *n* = 5.

**Table 5:** Types of interactions between the three main essential oil constituents and the telomerase target.

Ligand	Amino Acid	Ligand Atom	Interaction	Type	Distance	Score
Terpinolene	MET482	Ligand	Hydrophobic	Alkyl	5.27	49.15
	ILE550	Ligand	Hydrophobic	Alkyl	4.75	
	ARG486	C1	Hydrophobic	Alkyl	4.09	
	ILE550	C1	Hydrophobic	Alkyl	4.52	
	LEU554	C7	Hydrophobic	Alkyl	4.41	
	MET482	C8	Hydrophobic	Alkyl	4.20	
	ILE497	C8	Hydrophobic	Alkyl	4.22	
	LEU554	C8	Hydrophobic	Alkyl	4.65	
	PHE478	C8	Hydrophobic	Pi–Alkyl	5.27	
	PHE494	C1	Hydrophobic	Pi–Alkyl	4.60	
	PHE494	Ligand	Hydrophobic	Pi–Alkyl	3.88	
	TYR551	Ligand	Hydrophobic	Pi–Alkyl	4.96	
	TYR551	C7	Hydrophobic	Pi–Alkyl	5.19	
	TYR551	C8	Hydrophobic	Pi–Alkyl	4.76	
1,8-Cineole	LEU554	Ligand	Hydrophobic	Alkyl	4.96	34.01
	LEU554	C1	Hydrophobic	Alkyl	4.15	
	ARG557	C1	Hydrophobic	Alkyl	3.68	
	ILE497	Ligand	Hydrophobic	Alkyl	4.67	
	LEU554	Ligand	Hydrophobic	Alkyl	4.07	
	LEU554	C10	Hydrophobic	Alkyl	4.05	
*p*-Cymene	MET482	Ligand	Other	Pi–Sulfur	4.94	48.76
	PHE494	Ligand	Hydrophobic	Pi–pi Stacked	3.75	
	TYR551	Ligand	Hydrophobic	Pi–pi T-shaped	4.47	
	MET483	C1	Hydrophobic	Alkyl	5.47	
	ARG486	C1	Hydrophobic	Alkyl	4.44	
	ILE550	C1	Hydrophobic	Alkyl	4.26	
	LEU554	C8	Hydrophobic	Alkyl	4.89	
	PHE494	C1	Hydrophobic	Pi-Alkyl	4.66	
	PHE494	C8	Hydrophobic	Pi-Alkyl	4.83	
	TYR551	C8	Hydrophobic	Pi-Alkyl	5.11	
	ILE550	Ligand	Hydrophobic	Pi-Alkyl	5.04	

## Data Availability

Data is contained within the article.
